# Advances in Diagnostic Imaging Technologies to Evaluate the Retina and the Optic Disc

**DOI:** 10.1155/2015/371312

**Published:** 2015-06-29

**Authors:** Antonio Ferreras, Michele Figus, Paolo Frezzotti, Michele Iester

**Affiliations:** ^1^Department of Ophthalmology, Miguel Servet University Hospital, 50009 Zaragoza, Spain; ^2^University of Zaragoza, 50009 Zaragoza, Spain; ^3^Department of Neurosciences, University of Pisa, 56100 Pisa, Italy; ^4^Department of Ophthalmology, University of Siena, 53100 Siena, Italy; ^5^Clinica Oculistica, DINOGMI, University of Genoa, 16132 Genoa, Italy

Current diagnostic and therapeutic decisions based on the outcomes of imaging technologies have become a common practice in every ophthalmic subspecialty. New devices and tools for evaluating the retina and optic nerve head, such as scanning laser polarimetry and spectral-domain optical coherence tomography (OCT), are widely used in clinical practice. These technologies provide objective quantitative measurements and in vivo real-time images of ocular structures. The performance of imaging devices is continuously being improved, and thus knowledge of their applications, advantages, and limitations must also be continuously updated to optimize their management by clinicians.

While imaging technologies require relatively transparent media, the variability of the measurements acquired by these devices is low, making these instruments useful for monitoring changes over time. In this issue, M. Ara et al. report excellent reproducibility of scanning laser polarimetry in healthy and glaucoma patients. In their review article, J. J. Garcia-Medina et al. evaluated the effects of posterior capsule opacification and found that image quality improves after capsulotomy, but a new baseline for future comparisons should be established. Additionally, R. L. Brautaset et al. report that OCT allows for the acquisition of reliable macular measurements in individuals with moderate to severe keratoconus.

M. Cavallari et al. developed a semiautomated, computer-based method to detect and quantify retinal vessel abnormalities by analyzing digital fundus photographs. This tool was successfully used to evaluate patients with hypertensive retinopathy and cerebral autosomal dominant arteriopathy with subcortical infarcts and leukoencephalopathy.

G. S. K. Yau et al. compared central macular thickness measured by OCT in Chinese children and found that it was thicker in myopic eyes compared to emmetropic and hyperopic eyes.

Three papers regarding the role of the ganglion cell complex in different disorders, multiple sclerosis, age-related macular degeneration, and glaucoma, demonstrated that the same tool could be used with different approaches depending on the disease: “Comparative Diagnostic Accuracy of Ganglion Cell-Inner Plexiform and Retinal Nerve Fiber Layer Thickness Measures by Cirrus and Spectralis Optical Coherence Tomography in Relapsing-Remitting Multiple Sclerosis” by J. J. Gonzalez-Lopez et al.; “Can Variability of Pattern ERG Signal Help to Detect Retinal Ganglion Cells Dysfunction in Glaucomatous Eyes?” by A. Mavilio et al.; and “Ganglion Cell Complex Evaluation in Exudative Age-Related Macular Degeneration after Repeated Intravitreal Injections of Ranibizumab” by A. Perdicchi et al. J. J. Gonzalez-Lopez et al. observed a better sensitivity-specificity balance for the macular ganglion cell complex measured with OCT in relapsing-remitting multiple sclerosis than peripapillary retinal nerve fiber layer thickness, while A. Mavilio et al. found that reduction of the ganglion cell complex was related to reduced amplitude and increased variability of the phase of the steady-state pattern electroretinogram in glaucoma. On the other hand, A. Perdicchi et al. demonstrated that the loading phase for ranibizumab in aged-related macular degeneration had no toxic effects on the ganglion cell complex.

R. Mastropasqua et al. and M. Di Nicola et al. provide reviews regarding advanced morphologic and functional magnetic resonance techniques in glaucoma and functional and structural abnormalities in deferoxamine retinopathy, respectively. Information concerning magnetic resonance imaging in glaucoma is limited and mostly based on mouse models and patients with advanced glaucoma. Nevertheless, some results support the potential for these techniques to detect early glaucomatous changes. M. Di Nicola et al. revealed that imaging technologies (color fundus photographs, autofluorescence, and OCT) may facilitate the management of deferoxamine retinopathy and their report highlights the need for guidelines to enhance the diagnosis and follow-up of these patients.

A wide variety of image modalities and optic enhancements, including fundus autofluorescence, microperimetry, adaptive optics, or faster OCTs, recently emerged. Using different image modalities, clinicians can emphasize the features of a particular anatomic structure of a tissue. M. Bertolotto et al. evaluated various paracentral hyperautofluorescence retinal patterns and their relationship with changes in the retinal layers. Hyperautofluorescence was mainly related to a “window effect” rather than an accumulation of lipofuscin. Adaptive optics combined with OCT or fundus photography allow for high-resolution images due to the improvement in lateral resolution by correcting for aberrations in the eye. This technology reaches resolutions close to 2 to 4 *µ*m, which is high enough to identify even cone photoreceptors. D. Supriya et al. report strong correlations between retinal sensitivity, evaluated by microperimetry, and the mean cone packing density at different macular eccentricities measured with an adaptive optics retinal camera. Further studies are required, however, to determine normative variations in cone structure-function correlation.

OCT has changed many protocols and ways of managing different ocular diseases. Moreover, OCT has modified how clinicians look at the retina. The assessment of retinal abnormalities based on evaluation of every layer rather than the global thickness has advanced ophthalmology. Because images evaluated through OCT provide information of the actual retina anatomy, but not exactly the same information as obtained with histology; last year an international panel of experts in vitreoretinal diseases and imaging suggested a consensus nomenclature for the classification of retinal and choroidal layers and bands observed in OCT scans (IN•OCT consensus). P. Tortorella et al. evaluated the changes in two of these retinal layers, the photoreceptor inner segment ellipsoid band and the interdigitation zone, in eyes with uveitic macular edema. Interruption of these lines was related to poor visual acuity. R. L. M. Wong et al. report that the outer retinal layer thickness (distance between the external limiting membrane and retinal pigment epithelium) correlated better than total thickness with visual acuity in patients with diabetic macular edema.

In summary, this issue includes different points of view presented by diverse authors covering several topics related to advances in imaging techniques for ophthalmic diseases. This publication will provide valuable information that should be helpful in clinical practice.

*Antonio Ferreras* *Michele Figus* *Paolo Frezzotti* *Michele Iester*

